# Supplementary material for: Prevalence, patterns, and factors associated with tobacco use among patients with priority tobacco related illnesses at four Kenyan national referral hospitals, 2022
Source: PLOS Glob Public Health. 2023 Nov 10;3(11):e0002002. doi: 10.1371/journal.pgph.0002002 (PMC10637644; doi:10.1371/journal.pgph.0002002)
Supplement: Attachment — Submitted filename: Response to reviewers comments.docx [file pgph.0002002.s002.docx]

**Response to reviewers comments**

Reviewer #1: Overall, the manuscript is well written, and it focuses on an important public health issue, building on the existing evidence demonstrating the link between tobacco use and negative health effects. Being able to define and better understand the prevalence of use in this specific patient cohorts can help build a case for interventions for tobacco use prevention and cessation programs.

The authors can consider addressing the following comments to improve the clarity of the manuscript.

1. In the background section, the authors state "Furthermore, the findings on cost of health care for tobacco-related illnesses will help to inform stronger policy interventions that are required to minimize tobacco use and the resulting disease burden in Kenya." This is an overreach as the manuscript findings do not address any aspect of cost of health care, instead the findings focus on prevalence of tobacco use. The authors can consider including this costing aspect or editing this specific section of the background.

***Response****: we agree with the reviewer. This manuscript is part of three from the study, with the other two focusing on attribution and economic impact of health-related outcomes of tobacco use. Therefore, we have removed the reference to economic impact from this manuscript.*

2. In the background section, the authors end the section with the statement "Therefore, we sought to assess the prevalence of tobacco use among patients with at least one of four major TRI, at the four national referral hospitals in Kenya." The authors should spell out TRI on first use in the statement. Also, this is the first time the authors introduce four national referral hospital, leaving the reader lost. Consider describing the hospitals in this section or simply dropping "the".

***Response****: We agree with this observation and have corrected the section accordingly.*

3. In the methods section, the authors define the outcome variable of tobacco use well. It is not clear though whether information on duration of use and amount used (pack years) was collected? Even though for tobacco use there is no threshold for toxicity when it comes to health effects, I believe a better exposure evaluation is informative in building a case on the link between tobacco and TRIs and also in developing cessation programs in this specific cohort of patients. If collected this information can be included in the results section.

***Response****: We agree with this observation. Although not all this information is available, we have included average number of cigarettes per day and time to first use in the morning as indicators of magnitude of use and nicotine dependence.*

4. Table 3, tabulates the comparison between ever used tobacco and never used tobacco as it relates to the various TRIs, and table 4 shows factors associated with ever used tobacco in patients with tobacco related illnesses. While this is statistically sound, from a clinical perspective it is troublesome lumping an individual who has perhaps smoked one puff of a cigarette in their lifetime with one who has smoked 1 pack per day for a year. Was there any attempt made to measure the amount a person has smoked over a specified period of time?

***Response****: we agree with this observation. However, the decision to include all history of use, whether current or former, was informed by the fact that since our study population was people already diagnosed with serious health conditions, a substantial proportion have stopped use due to the diagnosis, therefore underestimating disease burden due to tobacco use. This is evidenced by the fact that we found 143 current users and 934 former users. However, we have enriched the results section with cessation findings.*

5. The authors state that reporting ever used tobacco was associated with increasing age. Wouldn't this naturally be expected with a measure of ever used.

***Response****: while this is true, the aim of the statement was to show that odds of tobacco use were higher among older TRI patients compared with younger ones. We have revised the sentence to depict this (also in the abstract).*

6. Perhaps for preventive programs a broad measure of ever used may be helpful, however for cessation program intervention, I believe current use measure is more critical in establishing burden and resource planning for cessation interventions.

***Response****: this is a correct observation. We have brought more results on current use and cessation into the manuscript.*

7. In the conclusion, the authors conclude with a statement "Our study highlights the real-world health implications of tobacco use in Kenya". This statement is an overreach as this was a cross-sectional survey showing associations and not causality. The statement suggests causality of TRIs was established in the study.

*Response: We agree with the reviewer. The sentence has been revised to read:* ***Our study highlights the burden of tobacco use among people diagnosed with priority TRI, as well as cessation and associations that can guide interventions..***

8. The authors can consider specifically including the need for cessation support programs for these cohort of patients in addition to anti-tobacco advocacy efforts mentioned in the conclusion.

***Response****: this has been included in all sections of the manuscript: abstract, results, discussion, and conclusion.*

9. Overall, the lack of granularity in the classification of tobacco use history misses the opportunity to better understand the burden and needs of these cohort of patients. The study authors merely classify use as current, former, and never without bringing out aspects of duration, frequency, and quantity of tobacco use.

***Response****: we have added the average number of cigarettes for smokers, as well as cessation, stratified among current users of smoked versus smokeless tobacco. The reason this has not been extended to the table 5 has been addressed above.*

10. Lastly, I believe there has already been great strides towards anti-tobacco advocacy and smoking cessation in Kenya and this has already translated in a lowered overall prevalence of tobacco use in the country. The authors can consider including this information in the background.

***Response****: we agree with the reviewer. We have referenced additional materials on the success Kenya has had in tobacco control in the introduction.*

Reviewer #2: Thank you for the opportunity to review this paper on the prevalence and factors associated with tobacco use among patients with tobacco-related illness in four national referral hospitals of Kenya. This is an important subject, particularly because it provides information for tobacco control policies in a lower-middle-income country with a high burden of tobacco use and limited treatment resources. My major concern is regarding the study's results, which predominantly confirm well-known findings in the existing literature. For instance, factors such as being female, married, having higher education, and having no history of alcohol use were already established as predictors of abstaining from tobacco use. However, the methods and the paper overall are very well written and may be more suitable for a local journal.

***Response****: we thank the reviewer and acknowledge this response. However, we feel that this study still complements what is already known, for two reasons. One, some factors like education level and period since diagnosis of TRI are timely and can guide packaging of cessations programs. Second, these results are part of a wide study that also included attributable mortality and health economic impact; the latter two manuscripts are also undergoing peer review. Read together, these three manuscripts, once published, will provide ample information for planning tobacco control programs.*

Reviewer #3: the data description, analysis and write up are up to standard according to my viewpoints .I am concerned that using a purposive sampling method for this type of data might make the results prone to bias,

***Response****: we agree with the reviewer that this could be a concern. However, the sampling was not overtly purposive. It was rather proportionately distributed among the four hospitals, and then all eligible patients recruited progressively when diagnosed. The reason for doing census was because some of the conditions are not diagnosed frequently in some of the hospitals. The positive side is we are confident we captured majority of people with the selected conditions, since these are managed only at referral hospitals in Kenya.*

Reviewer #4: The authors conducted a cross-sectional study linking tobacco use among patients with tobacco-related diseases. As such, there is no new information from this study. Similar published studies are already available (https://pubmed.ncbi.nlm.nih.gov/30455795/, https://pubmed.ncbi.nlm.nih.gov/30400915/). Rather, as the risk factors are already known, interventions around reducing tobacco use and demonstrating its effect would be more beneficial. Authors can try a regional journal for their manuscript.

***Response****: we thank the reviewer for the comment, but we beg to differ slightly. The suggested two articles, which are familiar to the current authors, were evaluating associations of tobacco use in the population, from two population-level surveys. Our study, however, examines the issue from the opposite angle; among those with conditions related to tobacco use, how much has history of tobacco use? What are their characteristics. What is their experience with cessation? We feel that this approach provides additional information, especially in the clinical component of tobacco control, that actually compliments rather than negates population-level control.*

Reviewer #5: Thank you for the study.

The title of the is confusing and does not provide a clear picture of the study conducted.

***Response****: the revised title now reads: Prevalence, patterns and factors associated with tobacco use among patients with priority tobacco related illness in at four Kenyan national referral hospitals, 2022*

Objectives of the study and justification for the study is not clear.

***Response****: this has now been clarified.*

Even though authors have collected a significant amount of data, they are not logically presented.

***Response****: this comment is not clear to us. The structure of the results move from socio-demographics to patterns of use, then cessation and lastly correlates.*

Discussion is very superficial and is mostly descriptive.

***Response****: We have enriched the discussion further.*

There is no meaningful comparison with the available literature, explanation's for findings and discussion.

***Response****: We have enriched the discussion further.*

Limitations of the study needs to be highlighted.

***Response***: *we have highlighted three key limitations of this study.*
